# Prevalence and associated factors of insomnia disorder among women attending antenatal care

**DOI:** 10.4102/sajpsychiatry.v31i0.2549

**Published:** 2025-10-10

**Authors:** Kennedy Kachingwe, Chimwemwe Munthali, Sylivester Malunga, Catherine Mselema, Geldine Chironda

**Affiliations:** 1 Department of Nursing and Midwifery, Faculty of Health Sciences, Saint John of God University, Mzuzu, Malawi; 2 Department of Clinical Medicine, Faculty of Health Sciences, Saint John of God University, Mzuzu, Malawi; 3 Seed Global Health, Lilongwe, Malawi

**Keywords:** insomnia disorder, pregnant women, antenatal care, Malawi, mental health disorder, sleep quality

## Abstract

**Background:**

Insomnia disorder is a modifiable risk factor for maternal and offspring psychopathology, yet it remains overlooked in low-resource settings like Malawi, where maternal mental health services are underprioritised.

**Aim:**

To assess the prevalence and associated psychiatric factors for insomnia disorder among pregnant women at Kamuzu Central Hospital (KCH).

**Setting:**

Antenatal care unit (ANC) at KCH, Lilongwe.

**Methods:**

A cross-sectional design was employed. Systematic random sampling was used to select 110 pregnant women. The Pittsburgh Sleep Quality Index (PSQI) was used to evaluate insomnia. Descriptive and logistic regression established the association between socio-demographic, obstetric, sleep arrangement and biometric factors and sleep quality.

**Results:**

The prevalence of insomnia disorder was found to be 79.1% (mean PSQI = 8.77 ± 3.79), indicating significant sleep disturbances. Logistic regression revealed a significant association with gestation, participants in the second trimester (adjusted odds ratio [AOR] = 5.21, 95% confidence interval [CI]: 1.49–18.26, *p* = 0.010) being at higher risk and increased odds among married women (odds ratio [OR] = 0.14, 95% CI: 0.13–0.48, *p* = 0.038) and those sharing bed (OR = 5.28, 95% CI: 1.14–24.55, *p* = 0.034) at higher risk of poor sleep quality.

**Conclusion:**

Poor sleep quality is common among pregnant women in Malawi, with trimester-specific and psychosocial predictors elevating psychiatric risks.

**Contribution:**

Integration of sleep screening and perinatal mental health interventions into antenatal care is urgently needed in the second trimester to mitigate adverse maternal and offspring outcomes.

## Introduction

Sleep quality during pregnancy is an essential yet overlooked aspect of maternal and offspring psychopathology.^1,2,3^ Approximately 40% – 62% of pregnant women globally report significant sleep disturbances with 57% in sub-Saharan Africa.^4,5,6,7^ Restorative sleep is vital for metabolic regulation, neuronal homeostasis and foetal development.^1,8,9^ However, pregnancy-related sleep disturbances arise from interplay of physiological and psychosocial factors that came along with gestation. Hormonal fluctuations characterised by progesterone-melatonin changes, physical discomforts (such as nocturia, back pain and respiratory challenges) and perinatal anxiety from ruminating over birth outcomes disrupt the sleep–wake cycle, elevating risk for neurodevelopmental and maternal mental health outcomes.^2,7,10,11^

Emerging evidence supports a bidirectional relationship between poor sleep and maternal mental health, with chronic sleep activating psychoneuroimmunological pathways contributing to psychiatric outcomes.^3,12,13^ These disturbances are linked to elevated cortisol from hypothalamic-pituitary-adrenal (HPA) axis dysregulation, increased pro-inflammatory cytokines (Interleukin-6 [IL-6], Tumour Necrosis Factor-alpha [TNF-α]) and altered neurotransmitter balance (Gamma-aminobutyric acid [GABA], serotonin, dopamine). These changes may impair amygdala-prefrontal cortex connectivity and emotional processing, creating intergenerational risks for neurodevelopmental disorders.^3,11,12,13,14,15^

Psychosocial factors, especially anxiety and depression, can disrupt sleep architecture by affecting stress-responsive neurotransmitters like serotonin 5-hydroxytryptamine (5-HT), reducing sleep duration and efficiency and contributing to postpartum depression.^16,17,18^ Maternal age also impacts sleep: younger women may have immature stress adaptation, while older women may experience insomnia related to age-associated obstetric risks.^19,20,21^ Elevated pre-pregnancy body mass index (BMI) exacerbates sleep issues through metabolic dysregulation and chronic inflammation.^20,22,23^ Similarly, maternal stress, social isolation, low perceived emotional support and late-night eating during pregnancy can further exacerbate sleep disturbances, especially as pregnancy progresses into the third trimester.^24,25,26,27^ These intersecting factors cumulatively increase risks for adverse maternal and child outcomes.

In low-resource contexts like Malawi, where socio-economic challenges are exacerbated by poverty and health disparities, there is a risk of elevated sleep disturbances that may be normalised and dismissed as inevitable. Factors such as poverty, Malawi’s prevalent malaria in pregnancy, intimate partner violence and maternal mental health illiteracy could exacerbate sleep disturbances during pregnancy.^3,28,29^ Despite evidence of pervasive lower back pain in pregnancy and high rates of adverse outcomes linked to poor sleep quality in Malawi,^30,31,32,33^ research has predominantly focused on depression and anxiety while neglecting the significance of sleep quality, a modifiable risk factor. The absence of targeted sleep screening in antenatal care at Kamuzu Central Hospital (KCH) emphasises the urgency for research examining sleep quality in this context. This study aimed at assessing the prevalence and associated factors of insomnia disorder among pregnant women attending antenatal care at KCH in Lilongwe, Malawi.

## Research methods and design

### Study design

The study adopted a cross-sectional, descriptive quantitative design.

### Setting

The study was conducted at the antenatal clinic of KCH, Lilongwe, the largest public tertiary healthcare facility in central Malawi. Kamuzu Central Hospital provides routine antenatal care (ANC) services to about 200 pregnant women monthly.

### Study population and sampling strategy

Participants were pregnant women attending routine ANC checkups at KCH. Inclusion criteria were being 1 month pregnant or above and consenting to participate. Exclusions included danger signs during pregnancy (vaginal bleeding, severe hypertension and hypotension), mental illness symptoms, preexisting insomnia, severe medical conditions and pregnant minors. The sample size was calculated as 110 from a population of 200 pregnant women attending ANC monthly.

Participants were selected using a systematic random sampling technique (random Kth interval generated by Excel). On each clinic day, 15 eligible participants were sampled from identification numbers in the waiting area. The population size (200) was divided by the sample size (110), resulting in a Kth interval of 1.82, rounded up to 2.

### Data collection

The Pittsburgh Sleep Quality Index (PSQI) was used, a self-rated questionnaire with 11 questions measuring the previous month’s seven sleep components: sleep duration, latency, efficiency, sleep medication use, daytime dysfunction, nocturnal disturbances and subjective sleep perception. Components are scored on a Likert scale of 0 to 3, where 0 is ‘no difficulty’, 1 is ‘less than once a week’, 2 is ‘once or twice a week’ and 3 is ‘three or more times a week’. The scores are summed to generate a global PSQI score from 5 to 21. A score > 5 indicates poor sleep quality consistent with DSM insomnia disorder symptoms and predicts insomnia.^34^ The PSQI is validated and has been used in low-resource sub-Saharan settings.^35,36^ Although the PSQI is conceptualised to measure sleep quality, for this study, it was used as a clinical diagnostic tool for insomnia disorder.

Relevant socio-demographic, socio-economic, obstetric, biometric information and sleeping arrangements were collected. Socio-demographic factors included age, religion, marital status, highest education level, monthly income, residence, domestic help availability, employment status, occupation and bed sharing. Obstetric factors included trimester, parity, gravidity and previous obstetric complications. Biometric factors included BMI and blood pressure. Participants with severe poor sleep were referred to mental health services.

### Data analysis

Data were coded, entered into SPSS version 20, cleaned and analysed. Independent variables included socio-demographic, obstetric and biometric factors. The dependent variable was sleep quality during the previous pregnancy month. Categorical variables were summarised with frequencies and percentages; continuous variables with means and standard deviations. Univariate analysis compared factors with sleep quality using chi-square tests. Multivariate logistic regression identified independent associations with poor sleep quality. Significance was set at *p* < 0.05 with 95% confidence intervals (CI).

### Informed consent

Participants received written information explaining the study’s benefits, risks, voluntary participation, withdrawal rights and confidentiality. All provided written consent before participation.

### Ethical considerations

Approval was obtained from the College of Medicine Ethics and Research Committee (COMREC) on 16 February 2024 (approval number P.11/23-0455). Procedures adhered to ethical standards of KCH, COMREC and the Helsinki Declaration.

## Results

All 110 questionnaires were retrieved and analysed, yielding a 100% response rate.

### Socio-demographic characteristics of participants

As shown in Table 1, the mean age of respondents was 28.47 ± 4.39 years. All had formal education; the majority identified as Christian (72.7%) and married (78.6%). Most resided in suburban areas (82.7%), and over half belonged to middle-income households (61.8%). Few reported no personal income.

**TABLE 1:** Socio-demographic variables of respondents (*N* = 110).

Variables	Frequency	%	Mean ± SD
**Age (years)**			28.47 ± 4.39
19–29	69	62.7	-
30–40	41	37.3	-
**Religion**			
None	-	-	-
Christian	92	83.6	-
Muslim	16	14.6	-
Other	2	2.8	-
**Educational status**			
Primary school	1	0.9	-
Secondary school	34	30.9	-
Tertiary school	75	68.2	-
**Marital status**			
Single	30	27.3	-
Married	80	72.7	-
**Occupation**			
Unemployed	36	32.7	-
Employed	30	27.3	-
Self-employed	44	40.0	-
**Asleep arrangement**			
No partner in bedroom	29	26.4	-
Does not share a bed	17	15.5	-
Shares bed	64	58.2	-
**Residence**			
Rural area	4	3.6	-
Suburban area	91	82.7	-
Urban area	15	13.7	-
**Monthly income**			
≤ K500000.00	12	10.9	-
> K500000.00–K100000.00	68	61.8	-
> K1 000000.00	30	27.3	-

SD, standard deviation.

### Obstetric characteristics of participants

A substantial proportion of participants (47.3%) were in their third trimester. Nearly half (49.1%) were primiparous, while among the multiparous, most reported no previous obstetric complication. Only 17% had BMI within the normal range.

### Prevalence of insomnia disorder

Using the PSQI, the overall prevalence of poor sleep quality indicative of insomnia was 79.1% (*n* = 87). The mean PSQI score was 8.77, accompanied by a standard deviation of ±3.792, indicating substantial clinically significant sleep disturbance.

Demographically, most with poor sleep quality (66.4%) were from suburban areas, and over half, 55.5% (*n* = 61) identified as Christians. Regarding age distribution, 47.3% (*n* = 52) fell within the 19–29 years. A significant proportion, 64.5% (*n* = 71), had a history of obstetric complications, and 43.2% (*n* = 48) classified as obese.

### Associations with socio-demographic, obstetric factors and sleeping arrangement

Logistic regression analyses revealed significant associations between sleep quality and covariates (Table 2).

**TABLE 2:** Logistic regression of factors associated with sleep quality among participants.

Variable	OR	95% CI	*p*	AOR	95% CI	*p*
Age	1.907	0.685, 5.313	0.217	-	-	-
Marital status	0.139	0.134, 0.483	**0.038**	2.491	0.427, 14.513	0.310
Education	0.663	0.255, 1.723	0.399	-	-	-
Christian	0.227	0.227, 1.589	0.302	-	-	-
Muslim	0.227	0.023, 8.829	0.602	-	-	-
Residence	0.342	0.080, 1.336	0.123	-	-	-
Unemployed	1.286	0.429, 3.852	0.654	-	-	-
Self-employed	1.370	0.437, 4.288	0.589	-	-	-
Domestic Help	1.035	0.485, 2.206	0.929	-	-	-
≤ K500000.00	0.909	0.193, 4.330	0.913	-	-	-
> K500000.00–K1000000.00	0.634	0.275, 2.197	0.777	-	-	-
Nulliparous	3.000	0.614, 14.652	0.175	-	-	-
Primiparous	3.937	0.764, 20.304	0.101	-	-	-
1st Trimester	0.223	0.068, 0.735	**0.014**	0.212	0.057, 0.790	**0.021**
2nd Trimester	0.253	0.073, 0.871	**0.029**	5.213	1.489, 18.260	0.10
Overweight	3.212	0.948, 10.882	0.491	-	-	-
Obese	1.568	0.436, 5.636	0.061	-	-	-
Obstetric complication	0.202	0.025, 1.608	0.131	-	-	-
Shares bed	5.283	1.137, 24.548	**0.034**	2.980	0.804, 11.047	0.102
Shares bedroom only	2.893	0.283, 1.137	0.274	-	-	-

Note: Bold *p*-values are significant at the 0.05 level of significance.

CI, confidence interval; OR, odds ratios; AOR, adjusted odds ratios.

Married women (*p* = 0.038, odds ratio [OR] = 0.139, 95% CI: 0.134–0.483), gestational age (*p* = 0.014, OR = 0.223, 95% CI: 0.068–0.735) and second trimesters (*p* = 0.029, OR = 0.253, 95% CI: 0.073–0.871) appeared as significant factors, showing reduced risk relative to the third trimester. Pregnant women who shared bed at night appeared more likely to have poor sleep quality (*p* = 0.034, OR = 5.283, 95% CI: 1.137–24.548). No significant associations were found with age, education, religion, residence, occupation, income, parity, BMI or prior obstetric complications (all *p* > 0.05).

In the multivariate analysis (Table 2), where confounding by gestational age, bed sharing and marital status was controlled, gestational age emerged as the sole predictor of sleep quality. First-trimester participants retained lower odds of poor sleep quality (*p* = 0.021, adjusted odds ratio [AOR] = 0.212, 95% CI: 0.057–0.790), while second-trimester participants showed significantly higher odds (*p* = 0.010, AOR = 5.213, 95% CI: 1.489–18.260), suggesting trimester-specific physiological or psychosocial stressors.

## Discussion

This study assessed insomnia disorder symptoms consistent with a PSQI score of less than 5, which signifies poor sleep quality than providing formal DSM diagnoses. The study reveals a concerning prevalence of insomnia disorder symptoms, represented with a prevalence of poor sleep quality of 79.1%, as high as figures reported in US,^37^ India,^38^ China^39^ and sub-Saharan Africa^35,36^ underscoring poor sleep quality in pregnancy as a global public health concern. The mean PSQI score of 8.77 at KCH indicates significant sleep disturbances, emphasising an area of perinatal mental health care that has been largely neglected yet poses risks of perinatal depression, anxiety disorders, suicidality and neurodevelopmental disorders in children overburdening families and the health care system.^10,11^

Women in their first trimester exhibited lower odds of poor sleep (AOR = 0.21), suggesting less hormonal or psychological impact during this period. However, those in the second trimester faced a fivefold increased risk (AOR = 5.21), identifying this stage as a critical window for intervention. Regional factors such as endemic malaria may further exacerbate sleep disturbances, emphasising the need for context-specific maternal care approaches that address both biological and psychosocial influences.

Interestingly, married women reported higher instances of poor sleep, possibly due to increased household responsibilities and caregiver demands, while bed-sharing was significantly associated with sleep disturbances (OR = 5.28). The presence of a partner may disrupt sleep through movement or snoring, especially during pregnancy.^40,41^

Despite the focus on insomnia disorder as a form of poor sleep quality rather than a clinical diagnosis, the study presents actionable insights. There is an urgent need to incorporate sleep health screenings into routine antenatal care practices. Educational initiatives focused on sleep hygiene for pregnant women and their partners could help mitigate the adverse effects of poor sleep on perinatal mental health and promote better outcomes for mothers and their children.

Limitations of this study include: The cross-sectional design, which prevents causal inference and cannot establish whether associated factors cause or result from insomnia. Sleep disturbances during pregnancy can vary across trimesters, and a single measurement may fail to capture these fluctuations. The exclusion of other sleep disorders, particularly obstructive sleep apnoea – especially given the high BMI in the sample – may have affected the accuracy of the ‘insomnia’ diagnosis. Additionally, reliance on self-reported PSQI scoring for poor sleep quality for a month, which does not fully capture insomnia disorder and has the potential to introduce bias and lurking psychological associated factors. Moreover, selection bias could have occurred due to the varied times of day and different trimesters, and a stratified sample might have minimised assumptions across trimesters.

## Conclusion

This study highlights the high prevalence of insomnia disorder, characterised by poor sleep quality in pregnancy, as a modifiable risk factor for perinatal mental health issues in Malawi. Focusing on sleep health during antenatal care, especially in the second trimester, can improve maternal well-being and child developmental outcomes in resource-limited settings. Referring participants with severe sleep disturbances to other health services provides an opportunity for further investigation. Future research should utilise longitudinal approaches with objective sleep assessments, such as actigraphy, and consider the influence of psychological factors to explore the intricate relationships between sleep quality and mental health in this vulnerable population.
